# Fast and Analytical EAP Approximation from a 4th-Order Tensor

**DOI:** 10.1155/2012/192730

**Published:** 2012-12-30

**Authors:** Aurobrata Ghosh, Rachid Deriche

**Affiliations:** ATHENA Research Team, INRIA Sophia Antipolis Méditerranée, 2004 Route des Lucioles, BP 93, 06902 Sophia Antipolis Cedex, France

## Abstract

Generalized diffusion tensor imaging (GDTI) was developed to model complex apparent diffusivity coefficient (ADC) using higher-order tensors (HOTs) and to overcome the inherent single-peak shortcoming of DTI. However, the geometry of a complex ADC profile does not correspond to the underlying structure of fibers. This tissue geometry can be inferred from the shape of the ensemble average propagator (EAP). Though interesting methods for estimating a positive ADC using 4th-order diffusion tensors were developed, GDTI in general was overtaken by other approaches, for example, the orientation distribution function (ODF), since it is considerably difficult to recuperate the EAP from a HOT model of the ADC in GDTI. In this paper, we present a novel closed-form approximation of the EAP using Hermite polynomials from a modified HOT model of the original GDTI-ADC. Since the solution is analytical, it is fast, differentiable, and the approximation converges well to the true EAP. This method also makes the effort of computing a positive ADC worthwhile, since now both the ADC and the EAP can be used and have closed forms. We demonstrate our approach with 4th-order tensors on synthetic data and in vivo human data.

## 1. Introduction

Generalized diffusion tensor imaging (GDTI) [1–3], was proposed to model the apparent diffusion coefficient (ADC) recovered by diffusion MRI (dMRI) when imaging the diffusion of water molecules in heterogeneous media like the cerebral white matter. Essentially, GDTI uses higher-order Cartesian tensors (HOTs) to model the spherical profile of the ADC. However, although the complex shape of the ADC reflects the complex geometry of the underlying tissue, it is well known that the geometry of the ADC does not correspond to the underlying fiber directions [4]. This can be understood from the *q*-space formalism, where it can be seen that the ADC and the diffusion signal are in the Fourier domain of the diffusion ensemble average propagator (EAP), which describes the probability of the diffusing particles. The geometry of the EAP is a direct indicator of the microstructure of the underlying tissue or fiber bundles.

But GDTI was proposed because it overcomes the limitation of diffusion tensor imaging (DTI) [5], which is inadequate for modelling the signal from regions with multiple fiber configurations. In such regions, the HOT that is used in GDTI can model the signal and the ADC with greater accuracy than the 2nd-order diffusion tensor. Therefore, the GDTI model has been of considerable interest and has seen various developments. In particular, a number of contributions were made to estimate 4th-order HOTs in GDTI under the constraint of a positive diffusion profile since negative diffusion is nonphysical. A ternary quartic parameterization was used in [6–8], while a Riemannian approach was proposed in [9]. Other sophisticated methods were also proposed recently to estimate arbitrary even order HOTs in GDTI with the positivity constraint. In [10] the authors relied on a parameterization based on tensor decomposition into a sum of squares, and in [11], the authors used conic programming approaches to achieve this.

The GDTI model was also used to develop “biomarkers” or scalar indices such as the generalized anisotropy (GA) and the scaled entropy (SE) from HOTs modelling the ADC [3]. Additional scalar measures—in the form of invariants—were also proposed for 4th-order Cartesian tensors in [12, 13]. Overall, the GDTI model in particular and the Cartesian tensors in general have evoked great interest and have been extensively explored in dMRI. Various tensor-based models other than GDTI have been advanced and many methods (including GDTI) using tensors have been proposed to leverage the Cartesian and the algebraic structure of HOTs. A comprehensive review can be found in [14]. These indicate the importance and usefulness of HOTs in dMRI.

However, in spite of the interest in HOTs, to describe complex shaped ADCs, the tissue microstructure can only be inferred from the shape of the EAP. But computing the EAP from the HOT model of the ADC in GDTI is not an easy task. In [2], the authors proposed a numerical fast Fourier transform scheme—to emulate diffusion spectrum imaging (DSI) [15] from GDTI—to estimate the EAP and to recover the underlying fiber directions. However, this method is computationally expensive, and although the numerical Fourier transform can compute the values of the EAP at desired points, it cannot compute a continuous and differentiable function which has great advantages. That is perhaps the reason why the GDTI approach has been overshadowed by other methods that estimate the EAP or its characteristics directly from the signal, such as orientation distribution function (ODF) from Q-ball imaging (QBI), persistent angular structure (PAS-MRI), diffusion orientation transform (DOT), and spherical deconvolution (SD) [16–20]. It is interesting to note that DOT also uses the GDTI model to analytically compute the EAP on fixed shells from the ADC. However, in DOT, the spherical harmonic (SH) basis is used to model the ADC instead of Cartesian tensors.

In this paper, we propose a modification to the original GDTI model under the *q*-space formalism, which allows us to compute a closed-form approximation of the EAP using Hermite polynomials. In this modified model, we still estimate HOTs from the signal, but these HOTs are used to describe the ADC over the entire *q*-space instead of just its spherical profile. We show that the approximated EAP from the modified GDTI model converges well to the true EAP, and that it allows us to recover the fiber directions of the microstructure correctly. Furthermore, in our modified GDTI model, when we use 4th-order tensors, we are still able to apply the constraint of a positive diffusion profile while estimating the HOT from the signal before computing the EAP approximation from the tensor. Finally since the solution is analytical, it is fast and can be implemented efficiently.

We first test this approach on a synthetically generated dataset that simulates crossing fibers. We compare the computation time of this method with a numerical discrete Fourier transform scheme to recover the EAP from the original GDTI model. We show that with our modified approach, we are able to recover the underlying fiber layout correctly and also gain considerably in computation time. This is of great relevance in visualization and in post-processing such as tractography. We also conduct experiments on *in vivo* human cerebral data to illustrate the applicability of this approach on real data. We find that the peaks of the approximate EAP clearly reveal major fiber bundles and also discern crossings in the white matter.

The rest of the paper is structured as follows. In Section 2, we present the main theory and mathematical formulation of the modified GDTI model and algorithm to estimate the EAP from the GDTI-HOT. We illustrate on 4th-order tensors. In Section 3, we describe the synthetic and *in vivo* data, describe the experiments, and present the results. In Section 4, we discuss some properties of the approximation and how the approximate EAP behaves with respect to the true EAP. We finally conclude in Section 5.

## 2. Materials and Methods

### 2.1. Modified GDTI

We recall that the signal *E*(**q**) = *S*(**q**)/*S*_0_, in GDTI [1], is modelled using a *k*th-order tensor *𝒟* ^(*k*)^ (with *k* being even), which describes the spherical or angular profile of the ADC:

(1) E(q)k=exp(−4π2q2t∑j1=13∑j2=13⋯‍∑jk=13Dj1j2⋯jk(k)gj1gj2⋯gjk)=exp(−4π2q2t∑m+n+p=kDmnp(k)g1mg2ng3p),

where **q** = *γδ* **G**/2*π* when **G** is the diffusion encoding gradient vector, *t* = (Δ − *δ*/3), and *g*_*j*_ are the components of the unit gradient vector **g** = **G**/|**G**|. The second equality is a reinterpretation of the first by a rearrangement of the indices that highlights the polynomial interpretation of tensors [6]. *k* = 4 gives the 4th-order diffusion tensor model. We also recall that in the *q*-space formalism, the diffusion signal and the EAP are related by the Fourier transform [21]:

(2) $$\begin{array}{c} P\left(r\right)=\int E\left(q\right)\exp\left(-2\pi iq^{T}r\right)dq. \end{array}$$

The *q*-space formalism entails the “pulsed” gradient condition that *δ* ≪ Δ, implying that *t* ≈ Δ.

For *k* = 2, *E*(**q**)_2_ is the DTI model, whose Fourier transform *P*(**r**)_2_ is well known to also be a Gaussian, which corresponds to the free diffusion propagator. We denote by *P*(**r**)_*i*_ the EAP computed from the Fourier transform of *E*(**q**)_*i*_. However, for general *k* > 2, closed forms for the Cartesian Fourier transform of *E*(**q**)_*k*_ are hard to compute, since in Cartesian coordinates, *E*(**q**)_*k*_ is not separable in *q*_1_, *q*_2_, and  *q*_3_, the components of **q**. In [2], where a method for recovering the EAP from GDTI is proposed, *P*(**r**)_*k*_ is computed numerically by evaluating *E*(**q**)_*k*_ more or less densely in *q*-space and by computing its fast Fourier transform.

In this section, we propose to modify the original GDTI model by making (1) separable in Cartesian coordinates. This is done by realizing that GDTI in fact uses two orders *k*1 and *k*2 for the radial and the angular components, respectively,

(3) E(q)k1,k2=exp(−4π2qk1αt∑m+n+p=k2Dmnp(k2)g1mg2ng3p)

(4) =exp(−4π2qk1−k2αt∑m+n+p=k2Dmnp(k2)q1mq2nq3p),

where in GDTI *k*1 = 2 and *k*2 = *k*. In this formulation, *α* is a constant with units *m* ^2−*k*1^ that makes the exponent unit free when *k*1 ≠ 2 and *q* = |**q**|. The first equality is written in the components of the unit gradient vector **g**, while the second equality is in the components of the reciprocal space vector **q**. To pass from the first to the second equality, the components of **g** have to be multiplied by *q*, the norm of **q**, raised to the appropriate power, *k*2. To leave the equality unchanged, it is therefore necessary to also multiply by *q* ^−*k*2^, which results in the second equality. This reformulation of GDTI allows *E*(**q**) to become separable in *q*_1_, *q*_2_, and  *q*_3_ when *k*1 = *k*2 = *k*.

Although the original formulation of GDTI uses a Cartesian HOT, it was in essence written in spherical coordinates since the HOT *𝒟* ^(*k*)^ was evaluated only along the unit gradient vector **g**. Alternately, the spherical coordinates also become evident from the two separate orders *k*1 and *k*2 for the radial and the angular parts. By equating the two orders *k*1 = *k*2, our modification converts the signal formulation to Cartesian coordinates and recouples the radial and the angular parts. The HOT is now evaluated over the entire *q*-space. This reformulation allows us to compute an analytical Fourier transform of *E*(**q**)_*k*,*k*_ in Cartesian coordinates.

Interestingly, in spite of this reformulation, the signal in (4) still retains a monoexponential form parameterized by the diffusion HOT *𝒟* ^(*k*2)^, like in the original formulation. In (1), the negative logarithm of the signal is *b* · ADC, where *b* = 4*π* ^2^ *q* ^2^ *t*. In the modified model, if we denote *b*′ = 4*π* ^2^ *αt*, then

(5) $$\begin{array}{c} \ln\left(E{\left(q\right)}_{k,k}\right)=-b^{\prime }\cdot \hat{\text{ADC}}=-b^{\prime }\sum_{m+n+p=k}D_{mnp}^{\left(k\right)}q_{1}^{m}q_{2}^{n}q_{3}^{p}. \end{array}$$

This makes it evident that we can again estimate the HOT *𝒟* ^(*k*)^ from the diffusion signal in such a way that its diffusion profile is positive, that is, $\hat{\text{ADC}}>0$. Here, the diffusion profile $\hat{\text{ADC}}$ is no longer a function on the sphere but rather a function on the entire *q*-space. However, from this equation, we also see that when *k* = 4, the methods that were developed to estimate a 4th-order tensor with a positive diffusion profile for the GDTI model in (1), can all be directly applied to the modified model in (4). Therefore, using the modified model in (4), it is possible to estimate a 4th-order HOT from the signal which satisfies a positive diffusion profile, before computing the EAP from this HOT. In this paper, we use the ternary quartic parameterization proposed in [8].

### 2.2. Fast and Analytical EAP Approximation

Our solution for the EAP from the modified GDTI model pivots around the following property of the Fourier transform:

(6) $$\begin{array}{c} ℱ\left\{x^{n}f\left(x\right)\right\}={\left(\frac{i}{2\pi }\right)}^{n}\frac{d^{n}}{dt^{n}}ℱ\left\{f\left(x\right)\right\}\left(t\right), \end{array}$$

where *ℱ* stands for the Fourier transform. If we employ *g*(*x*) = *e* ^−2*π*^2^*x*^2^^ for *f*(*x*), then its Fourier transform is $G\left(t\right)=ℱ\left\{g\left(x\right)\right\}\left(t\right)=(1/\sqrt{2\pi })e^{-t^{2}/2}$. However, the derivatives of the Gaussian function *G*(*t*) generate the Hermite polynomials (−1)^*n*^(*d* ^*n*^/*dt* ^*n*^)*e* ^−*t*^2^/2^ = *He*_*n*_(*t*)*e* ^−*t*^2^/2^. Therefore,

(7) $$\begin{array}{c} ℱ\left\{x^{n}e^{-2\pi^{2}x^{2}}\right\}\left(t\right)={\left(\frac{-i}{2\pi }\right)}^{n}He_{n}\left(t\right)\frac{1}{\sqrt{2\pi }}e^{-t^{2}/2}. \end{array}$$

The generalization to 3D is simple since the Gaussian function is separable in the variables.

To take advantage of this property of the Fourier transform, and those of the Gaussian function, for computing a closed-form approximation of the EAP from *E*(**q**)_*k*,*k*_, that is, its Fourier transform *P*(**r**)_*k*,*k*_, we propose to expand *E*(**q**)_*k*,*k*_ as a multivariate polynomial multiplied by a 3D Gaussian function:

(8) $$\begin{array}{c} E{\left(q\right)}_{k,k}\approx \left(\sum C_{l,s,u}q_{1}^{l}q_{2}^{s}q_{3}^{u}\right)\exp\left(-2\pi^{2}\beta \left(q_{1}^{2}+q_{2}^{2}+q_{3}^{2}\right)\right), \end{array}$$

where *β* is a constant with units m^2^ to render the exponent unit free, and the new coefficients *C*_*l*,*s*,*u*_ in the polynomial expansion contain the imaging parameter *b*′ and the coefficients of the HOT *𝒟* ^(*k*)^. If this expansion was possible, then *E*(**q**)_*k*,*k*_ would become separable in *q*_1_, *q*_2_, and  *q*_3_.

Such an expansion can be achieved from a few manipulations and a Taylor expansion:

(9) E(q)k,k=exp((−4π2αt∑Dmnp(k)q1mq2nq3p)     +2π2β(q12+q22+q32))×exp(−2π2β(q12+q22+q32))=h(q)exp(−2π2β(q12+q22+q32)),

where the summation in the first equality is over *m*, *n*, and  *p* such that *m* + *n* + *p* = *k*, as denoted in (4), and *h*(**q**) = exp((−4*π* ^2^ *αt*∑*D*_*mnp*_ *q*_1_^*m*^ *q*_2_^*n*^ *q*_3_^*p*^) + 2*π* ^2^ *β*(*q*_1_^2^ + *q*_2_^2^ + *q*_3_^2^)). Since *h*(**q**) is an exponential function *e* ^*X*(**q**)^, we define *h*_*n*_(**q**) as the *n*th-order Taylor expansion of *h*(**q**) in the variables *q*_1_, *q*_2_, and  *q*_3_. Therefore, *h*_*n*_(**q**) is a trivariate polynomial of degree *n* − 1 plus an error term of degree *n*. Ignoring the error term, *h*_*n*_(**q**) has the required form *h*_*n*_(**q**) = ∑_*l*+*s*+*u*<*n*_ *C*_*l*,*s*,*u*_ *q*_1_^*l*^ *q*_2_^*s*^ *q*_3_^*u*^. Therefore, we can define the *n*th-order approximation of the signal:

(10) E(q)k,k(n)=hn(q)exp(−2π2β(q12+q22+q32))=(∑l+s+u<nCl,s,uq1lq2sq3u)exp(−2π2β(q12+q22+q32)).

Since *h*_*n*_(**q**) is the Taylor's expansion of an exponential function *h*(**q**), *h*_*n*_(**q**) converges to *h*(**q**) uniformly over all **R** ^3^ as *n* is made large. Therefore, *E*(**q**)_*k*,*k*_^(*n*)^ converges to *E*(**q**)_*k*,*k*_ uniformly over **R** ^3^ as *n* is made large.

As *E*(**q**)_*k*,*k*_^(*n*)^ is separable in *q*_1_, *q*_2_, and  *q*_3_, it is possible to compute a closed form for its Cartesian Fourier transform, which is also separable. Using the property in (7):

(11) P(r)k,k(n)=1(2πβ)3/2exp(−12β(r12+r22+r32))×(∑l+s+u<n(−i2π)l+s+uCl,s,uHel(r1)Hes(r2)Heu(r3)).

For large *n*, the approximation *P*(**r**)_*k*,*k*_^(*n*)^ converges to the true EAP *P*(**r**)_*k*,*k*_. In practice, we use *n* = 5,7, 9.

We thus find a closed-form approximation of the EAP from the modified GDTI model of the ADC using HOTs. The solution is a polynomial multiplied by a Gaussian. Therefore, the polynomial can be interpreted as the correction to the free diffusion Gaussian EAP due to the complex heterogeneous medium.

An alternate interpretation to this method can be found from (10) and (11), which avoids modifying the GDTI model. Equations (10) and (11) resemble closely the formulation of the signal in the expansion of the cumulant generating function, and the approximation of the EAP using the Gram-Charlier series, as proposed in [22]. While in [22], in the cumulant expansion, the signal is expanded in the standard polynomial basis with the cumulants as the coefficients, in (10) the signal is in fact expanded in a subset of the standard polynomial basis. Since the Fourier transform of a monomial multiplied by a Gaussian is a Hermite polynomial multiplied by a Gaussian, in [22] too, the EAP is approximated in the Hermite polynomial basis—again with the cumulants as the coefficients using the Gram-Charlier series. Likewise in (11), the EAP is approximated in the Hermite polynomial basis.

The difference between this method and [22] lies in the fact that while [22] uses the entire polynomial (Hermite polynomial) basis to expand the signal (EAP), this method uses only a subset of these bases. Therefore, the coefficients *C*_*l*,*s*,*u*_ are no longer the cumulants. Or, in other words, if the entire polynomial basis had been used here, then *C*_*l*,*s*,*u*_ would have become the cumulants. Also, the coefficients *C*_*l*,*s*,*u*_ are not estimated directly from the signal, though they can be if (10) were used, but *C*_*l*,*s*,*u*_ are instead computed from the coefficients of the tensor *𝒟* ^(*k*)^ and the Taylor expansion in (10). Therefore, changing the order *n* of the approximation has an effect on the approximated EAP, since it adds or subtracts terms in (11). However, as shown in the experiments, this does not affect the direction of the peaks of the approximate EAP.

We program an efficient implementation of the proposed method through symbolic computation. Using Maple and assuming (4), we expand *E*(**q**)_*k*,*k*_ into a Taylor series in the variables *q*_1_, *q*_2_, and  *q*_3_ up to predefined orders *n* = 5,7, 9. This expansion automatically computes for us the new coefficients *C*_*l*,*s*,*u*_ from the coefficients of the HOT *𝒟* ^(*k*)^ (10). The EAP approximation *P*(**r**)_*k*,*k*_^(*n*)^, is then generated by again computing the Fourier transform of *E*(**q**)_*k*,*k*_^(*n*)^ symbolically. The expansion of the EAP is then converted to C-code using Maple, which is compiled. This routine therefore takes the imaging parameters as input, namely, *t* and the coefficients of *𝒟* ^(*k*)^ that are estimated from the diffusion signal. *α*, *β* are taken to be equal to 1.

## 3. Experiments and Results

Although we developed the theory for arbitrary *k* = *k*1 = *k*2, for the following experiments we consider *k* = 4, that is, *E*(**q**)_4,4_ and Pr_4,4_^(*n*)^. This is because, as we have seen, for *E*(**q**)_4,4_, we can employ the estimation techniques that guarantee that the 4th-order HOT has a positive diffusion profile, $\hat{\text{ADC}}>0$. In all the following the 4th-order HOT *𝒟* ^(4)^ is estimated from (3), with *k*1 = *k*2 = 4 using the method described in [8]. The estimation in [8] is described for (1), which depends on the *b*-value, that is, *b* = 4*π* ^2^ *q* ^2^ *t*. We adapt this to (3) by replacing the *b*-value by the imaging parameters 4*π* ^2^ *αt* and the ADC by the $\hat{\text{ADC}}$. We test the approach first on synthetic data and then on *in vivo* human cerebral data.

### 3.1. Synthetic Dataset

To conduct controlled experiments with known ground truths, we use a multitensor approach to generate synthetic DWIs [23]. The EAP corresponding to a single fiber is taken to be an anisotropic free diffusion Gaussian distribution, parameterized by a covariance tensor *𝒟* = diag(1390,355,355) × 10^−6^ mm^2^/s in its canonical coordinates. *𝒟* is rotated using rotation matrices to orient the fiber in space. We generate the signal DWIs for the single fiber by considering the *q*-space formalism and taking the Fourier transform of the Gaussian EAP, which results in the anisotropic Stejskal-Tanner signal equation. Multiple crossing fibers are simulated by considering an EAP, that is, the weighted sum of free diffusion Gaussians, where each Gaussian represents a fiber oriented in space. The signal DWIs for a multifiber or crossing fiber is derived easily in the same fashion as *S*(**g**_*i*_) = ∑_*k*=1_^*N*^ *w*_*k*_ *e* ^−*b ***g**_*i*_^*T*^*𝒟*_*k*_**g**_*i*_^, such that ∑_*k*_ *w*_*k*_ = 1, *𝒟*_*k*_ = **R**_*k*_^*T*^ *𝒟* **R**_*k*_ with **R** a rotation matrix, *S*(**g**_*i*_) represents the DWI along the *i*th gradient direction, and *N* are the number of fibers crossing in the voxel. We use a *b*-value of 3000 s/mm^2^ to generate the signal and corrupt it with a Rician noise with signal to noise ratio (SNR) of 30. The gradient directions are considered isotropically spread out on the sphere along 81 encoding directions. Since the dataset is generated from a fixed *b*-value, we consider the imaging parameter *t* = 50 ms, which allows us to compute *q*.

### 3.2. *In Vivo* Human Cerebral Dataset

The *in vivo* human cerebral dataset, described in [24], was acquired with a whole-body 3T Siemens Trio scanner, with an 8-channel array head coil and maximum gradient strength of 40 mT/m. The DWIs were acquired using spin-echo echo planar imaging (EPI) (time repetition [TR] = 12 s, echo time [TE] = 100 ms, 128 × 128 image matrix, FOV = 220 × 220 mm^2^, 72 slices with 1.7 mm thickness (no gap) covering the whole brain). The diffusion weighting was isotropically distributed along 60 encoding directions, with a *b*-value of 1000 s/mm^2^. Seven images without any diffusion weightings were placed at the beginning of the sequence and after each block of ten DWIs as anatomical reference for offline motion correction. Random variations in the data were reduced by averaging 3 acquisitions, resulting in an acquisition time of about 45 minutes. The SNR in the white matter of the *S*_0_ image was estimated to be approximately 37. The motion correction for the DWIs was combined with a global registration to T1 anatomy images. The gradient direction for each volume was corrected using rotation parameters. The registered images were interpolated to the new reference frame with an isotropic voxel resolution of 1.72 mm.

### 3.3. Synthetic Dataset Experiment

**Figure 1:**
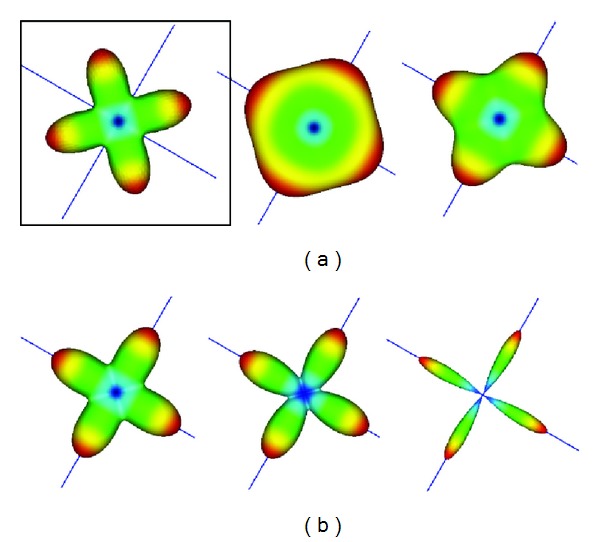
Spherical profiles of (a) the $\hat{\text{ADC}}$ estimated from the modified GDTI with a 4th-order tensor and (b) EAP approximation, *P*(**r**)_4,4_^(7)^, with increasing |**r**| from 12 *μ*m to 20 *μ*m.

A first example of the synthetic data and the results of our method are shown in Figure 1. In this proof of concept experiment, we consider two fibers crossing perpendicularly in a voxel with equal weights. On the left, in Figure 1(a), is shown the positive $\hat{\text{ADC}}$ modelled by a 4th-order tensor which was estimated using [8]. In Figure 1(b) are shown the profiles of the analytically approximated EAP (*n* = 7) for increasing norms of the vector **r** from 12 *μ*m to 20 *μ*m. As expected, we observe the desired “sharpening” effect from the different profiles of the EAP as the probability of water molecules diffusing declines sharply along nonfiber directions as the radius of the probability distribution is increased. This provides a strong motivation for estimating positive higher-order diffusion tensors from the (modified) GDTI model, since now from the analytically approximated EAP it is also possible to infer underlying fiber directions.

In the main synthetic data experiment, we consider two fiber bundles crossing or overlapping in a way that makes them converge and diverge. This changes their crossing angles gradually in the region where they intersect. The voxels outside the fiber bundles are generated using an isotropic diffusion profile. We set three goals for this experiment. First, we test if our analytically approximate EAP can recover the three types of voxel models from the noisy DWIs, namely, isotropic, single fiber, and crossing fiber voxels. Second, we compare the computation time of our proposed method to the numerical Fourier transform approach. Finally, third, we also conduct tests on the effects of the estimation order on the EAP; these are discussed in Section 4.

**Figure 2:**
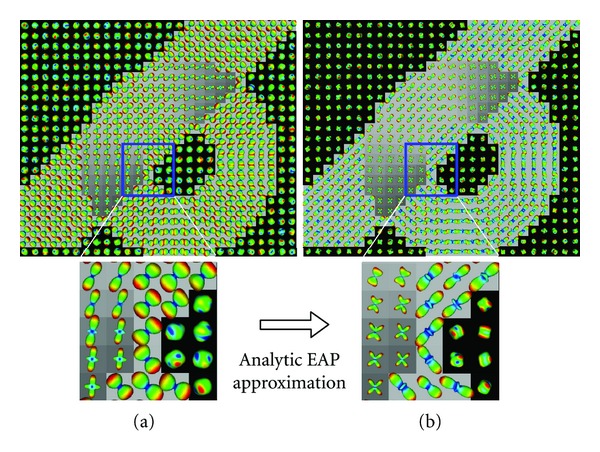
Synthetic dataset experiment. Two fiber bundles intersecting with the DWI signal corrupted by a Rician noise of SNR = 30. (a) $\hat{\text{ADC}}$ from 4th-order tensors. (b) Analytically approximated EAP, Pr_4,4_^(7)^.

The layout of the synthetic dataset fibers and the result of the estimated $\hat{\text{ADC}}$ from 4th-order HOTs and the analytical EAP approximations of order 7, Pr_4,4_^(7)^ are presented in Figure 2. It shows the two simulated fiber bundles and the three types of voxel models that constitute the synthetic dataset experiment. The effect of the EAP transformation of the $\hat{\text{ADC}}$ are highlighted in the zooms. Although the $\hat{\text{ADC}}$ profiles clearly indicate the regions with complex microstructures, that is, fiber crossings, the geometry of the $\hat{\text{ADC}}$ is not aligned with the fiber bundle directions. The peaks of the EAP on the other hand correctly indicate the underlying fiber directions.

**Figure 3:**
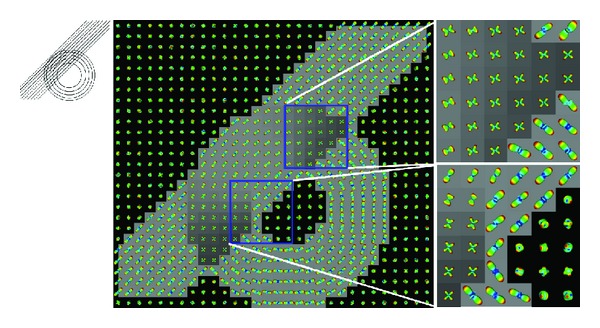
Synthetic dataset experiment. Left: fiber bundle layout. Centre: Pr_4,4_^(7)^. Right: zoom into the regions with crossings—top: the changing angle between the fiber bundles is detected and bottom: three types of voxels—isotropic, single fiber, and crossing fibers.

To evaluate the validity of the EAP approximation, we have proposed the angular profiles of the EAP, for fixed |**r** | = 20 *μ*m that are presented in Figure 3. In the two zooms, we take a closer look at some of the voxels in the crossing regions. In the top zoom, we see that the peaks of the angular profile of the EAP correctly detect the changing angle between the converging or diverging fiber bundles. In the bottom zoom, we see the three different types of voxels recovered by the approximate EAP, namely, the isotropic, the single fiber, and the two fibers crossings. Although the isotropic voxels also have some peaks, the peaks of the EAPs representing crossings are much more sharp, and it is easy to distinguish these two types of voxel geometries. The peaks in the isotropic voxels are caused by the signal noise and augmented order (4th) of the HOT. Nonetheless, as opposed to many other higher-order models such as PASMRI or SD, the voxels not containing fiber bundles are clearly identifiable as isotropic.

Speed is of great relevance in visualization and in processing after local estimation, such as in tractography. The closed-form of Pr_4,4_^(*n*)^ makes it computationally efficient, especially since the expression for a fixed *n* can be hard coded and compiled. In the synthetic data experiment, we compare this approach to a numerical Fourier transform of the GDTI model. For visualization and comparison, we consider the whole slice, which is partially seen in Figure 3, with 30 × 30 voxels. For the implementation of the numerical Fourier transform, we evaluate the GDTI model (1) on a 21 × 21 × 21 Cartesian grid. We evaluate the numerically computed EAP on a coarse spherical mesh with 162 vertices. The results are presented in Table 1. The computation time on our computer was 526 s. We then compute Pr_4,4_^(7)^, but this time on a finer spherical mesh with 2562 vertices. The computation time on the same computer was 73 sec. Despite the finer mesh, Pr_4,4_^(7)^ is about seven times faster than the regular discrete Fourier transform. On the coarse mesh with 162 vertices, the computation time for Pr_4,4_^(7)^ was about 10 s.

### 3.4. In Vivo Dataset Experiment

For the *in vivo* human cerebral dataset described above, we make certain assumptions about the imaging parameters since this dataset was acquired using a twice refocused Reese sequence [25], and not a standard pulsed-gradient spin-echo (PGSE) sequence. The gradient durations used in the Reese sequence were *δ*_1_ = 12.03 ms, *δ*_2_ = 19.88 ms, *δ*_3_ = 21.76 ms, and *δ*_4_ = 10.15 ms. As suggested in [26], Reese sequence parameters are sometimes adapted to the standard PGSE parameters with *δ* = *δ*_1_ + *δ*_2_, and Δ as the time between the start of *δ*_1_ and the start of *δ*_3_. However, since the application times of *δ*_*i*_ were unknown, we assume that *q* ^2^ = *b*, which implies 4*π* ^2^ *t* = 1.

**Figure 4:**
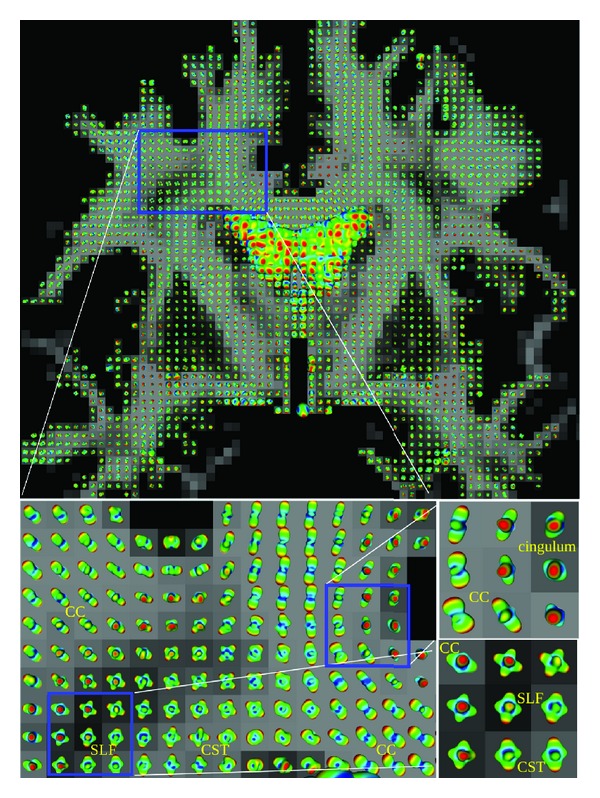
Real dataset experiment. A coronal slice with the Pr_4,4_^(7)^. The main zoom contains regions where three fiber bundles, namely, the CC, the CST, and the SLF intersect. The upper secondary zoom highlights the crossing between the CC and the cingulum due to partial voluming. The lower secondary zoom shows the main voxels with three peaks that correspond to the crossing between the CC, the CST, and the SLF.

We choose a coronal slice from the *in vivo* dataset where three fiber bundles are known to cross. The resulting analytically approximated EAPs from 4th-order tensors in the coronal slice are shown in Figure 4. In the plane horizontally and diagonally is the corpus callosum (CC), top to bottom is the corticospinal tract (CST), and going through the plane is the superior longitudinal fasciculus (SLF). The 4th-order HOTs were approximated from this dataset. In Figure 4, are shown the estimated order 7 approximations Pr_4,4_^(7)^ of the EAP. The zooms highlight the crossings between the major fiber bundles. In the main zoom is the region where the three fibers, the CC, the CST, and the SLF, intersect each other. In the upper secondary zoom, the crossing between the CC and the cingulum is highlighted, which occurs due to partial voluming. In the lower secondary zoom, are seen the main voxels with three peaks, which correspond to the crossing between the three fiber families—the CC, the CST, and the SLF.

## 4. Discussion

From the synthetic dataset and *in vivo* dataset experiments above, we were able to show that it is possible to recover the microstructure or fiber directions of the underlying tissue from our proposed analytical approximation of the EAP that was computed from 4th-order tensors. However, it is important to realize that the proposed approach only approximates the true EAP up to the truncation order of the Taylor series in (10). In this section, we validate the effects of this truncation or approximation on the fiber directions estimated by the approximate EAP. To do this, we again consider the synthetic dataset and look at a region where all three kinds of voxel models are present. We then compute the analytical EAP for approximation orders of *n* = 5,7, 9. We compare the angular profiles of these different approximations at a fixed radius |**r**| to the angular profiles of the fixed approximation at *n* = 7 for varying radii.

**Figure 5:**
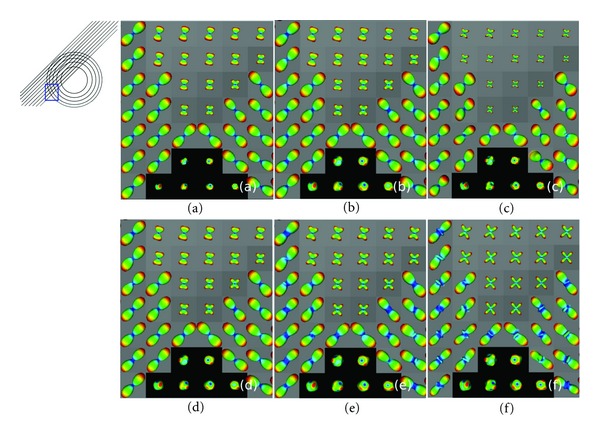
Effects of the approximating order *n* and the probability radius |**r**|. In top row, |**r**| is fixed, and we vary *n*. In bottom row, *n* is fixed, and we vary |**r**|. Top row: |**r** | = 16 *μ*m: (a) Pr_4,4_^(5)^, (b) Pr_4,4_^(7)^, and (c) Pr_4,4_^(9)^. Bottom row: *n* = 7  (Pr_4,4_^(7)^): (d) *≡* (b) |**r** | = 16 *μ*m, (e) |**r**| = 18 *μ*m, and |**r** | = 20 *μ*m.

Figure 5 shows the effect of the Taylor expansion order *n* on the EAP approximation. The six images are zooms into a region where the two fiber bundles converge and cross. In the top row, we present Pr_4,4_^(*n*)^, with *n* = 5,7, 9 evaluated for the probability radius |**r** | = 16 *μ*m. Increasing *n* adds more terms to the EAP approximation in (11), which adds more corrections to the approximation, making it converge better to the true EAP. As the approximation Pr_4,4_^(*n*)^, is corrected, it shows sharper peaks and narrower crossings for *n* = 9, than *n* = 5, for the same probability radius. However, this also increases the computation time. But the peaks of the lower-order approximations seem to be well aligned with the higher-order approximations. In other words, the peaks maintain their angular alignment, although they lose sharpness, and the EAP loses angular resolution, and narrow crossings become harder to discern. However, the angular resolution can be recovered, and the peaks “sharpened” in the lower-order approximations by increasing the probability radius, which saves computing time. This is shown in the bottom row, where we show Pr_4,4_^(7)^ for the probability radius varying from |**r**| = 16 *μ*m–20 *μ*m These experiments reveal that the effect of the Taylor expansion order *n* is to underestimate the EAP in the approximation. Therefore, we use the order 7 approximation Pr_4,4_^(7)^, as a good trade-off between convergence to the true EAP and computation time.

## 5. Conclusion

GDTI was developed to model complex ADC profiles which was an inherent shortcoming of DTI. GDTI uses HOTs of order *k* to model a complex ADC geometry. However, the shape of the ADC does not correspond to the underlying fiber directions. The microstructure of the tissue can be inferred from the geometry of the EAP, where in the *q*-space formalism, the EAP and the diffusion signal are related by the Fourier transform. But it is not easy to compute the EAP, *P*(**r**)_*k*_, from the HOT model of the signal *E*(**q**)_*k*_ in GDTI.

We overcome this hurdle by modifying the ADC model of GDTI, which allows us to approximate *E*(**q**) by a multivariate polynomial approximation and by proposing a novel closed-form approximation of *P*(**r**) using Hermite polynomials. The solution is a polynomial times a Gaussian; therefore, the polynomial can be interpreted as the correction to the Gaussian EAP due to the inhomogeneous medium. An alternate explanation can be used to explain this method, where the signal is expanded in the polynomial basis, and the EAP is expressed in the Hermite polynomial basis, which establishes the similarity of the proposed method to [22]. Also, since the solution is analytical, it is fast, and the approximation converges well to the true EAP.

In case of an order 4 HOT, this method can be directly adapted to the methods proposed for estimating 4th-order diffusion tensors with positive diffusion profiles. Therefore, it is possible to estimate a 4th-order HOT with a positive diffusion profile using this modified model before approximating the EAP. The experiments show that estimating only the 15 coefficients of a 4th-order HOT are enough to reveal the underlying fiber bundle layout. However, this is dependent on the order of the Taylor expansion used. Although the order of the expansion does not change the angular alignment of the peaks of the approximate EAP, it does affect its angular resolution or its capability of discerning narrow crossings. Increasing of the order increases the corrections to the approximation, which improves this angular resolution. However, it also increases the computation time. The angular resolution can be recovered in lower-order approximations, by increasing the probability radius, which saves computation time. However, this overall effect indicates that the truncation in the Taylor expansion has the effect of underestimating the true EAP in the approximation.

## Figures and Tables

**Table 1:** Computation time. A 30 × 30 voxel slice of the synthetic dataset was used for computations. The numerical Fourier Transform was performed on a coarse spherical mesh with 162 vertices to compute the angular profile of the EAP. The proposed analytical approximate EAPs were computed on both the coarse mesh and a finer spherical mesh with 2562 vertices. The analytical formulation is clearly advantageous.

	Numerical coarse	Analytical coarse	Analytical fine
Time	526 s = 8 m 46 s	10 s	73 s

## Abbreviations

CC: Corpus callosum
CST: Corticospinal tract
DOT: Diffusion orientation transform
DSI: Diffusion spectrum imaging
DTI: Diffusion tensor imaging
DWI: Diffusion-weighted image
EAP: Ensemble average propagator
GA: Generalized anisotropy
GDTI: Generalized DTI
HOT: Higher-(than 2) order (Cartesian) tensor
QBI: Q-ball imaging
ODF: Orientation distribution function
PASMRI: Persistent angular structure MRI
PGSE: Pulsed-gradient spin echo
SD: Spherical deconvolution
SE: Scaled entropy
SH: Spherical harmonic (basis)
SLF: Superior longitudinal fasciculus.
